# Immune response in adverse reactions to metal debris following metal-on-metal total hip arthroplasty

**DOI:** 10.1186/s12891-016-1069-9

**Published:** 2016-05-21

**Authors:** Masahiro Hasegawa, Takahiro Iino, Akihiro Sudo

**Affiliations:** Department of Orthopaedic Surgery, Mie University Graduate School of Medicine, 2-174 Edobashi, Tsuv, Mie 514-8507 Japan

**Keywords:** Adverse reaction to metal debris, Metal hypersensitivity, Metal-on-metal, Total hip arthroplasty

## Abstract

**Background:**

The purpose of the present study was to determine whether T cell-mediated type IV hypersensitivity reactions could be a major cause of adverse reaction to metal debris (ARMD) after metal-on-metal total hip arthroplasty (THA).

**Methods:**

Thirteen patients (1 man and 12 women; mean age 68 years, age range 60 to 83 years) with ARMD underwent revision surgery following metal-on-metal THA (15 hips). Lymphocyte stimulation testing was conducted. Periprosthetic tissue specimens underwent immunohistochemical studies.

**Results:**

Lymphocyte stimulation testing showed that five patients were nickel-sensitive, and one patient was also cobalt-sensitive. Immunohistochemical studies showed that T cells were dominant in five hips, and B cells were dominant in 10 hips. In four of the five patients with a positive lymphocyte stimulation test, the dominant lymphocytes were T cells, suggesting type IV hypersensitivity. The major cause of ARMD was not type IV hypersensitivity in the remaining nine patients.

**Conclusion:**

Metal hypersensitivity does not appear to be the dominant biological reaction involved in the occurrence of ARMD.

**Electronic supplementary material:**

The online version of this article (doi:10.1186/s12891-016-1069-9) contains supplementary material, which is available to authorized users.

## Background

Metal-on-metal total hip arthroplasty (THA) and hip resurfacing offer the theoretical advantages of decreased wear compared with polyethylene [1] and improved functional outcomes, but concern has emerged about metal-associated complications [2–6]. Metal-associated complications after metal-on-metal bearings are referred to as pseudotumors [2, 3]; Pandit et al. [2] showed that a soft-tissue mass associated with the implant was defined as a pseudotumor. Willert et al. [4] reported the pathologic term ALVAL (aseptic lymphocyte-dominated vasculitis-associated lesion). The characteristics of ALVAL are diffuse and perivascular infiltrates of T and B lymphocytes and plasma cells, many endothelial venules, massive fibrin exudation, accumulation of macrophages with drop-like inclusions, and infiltrates of eosinophilic granulocytes and necrosis. Histological evaluation of the changes in the periprosthetic tissues of patients who underwent revision for ALVAL suggested a cell-mediated type-IV hypersensitivity reaction (delayed-type hypersensitivity). It has been proposed that the cause of ALVAL could be metal hypersensitivity [4]. Langton et al. [5] identified that there is no clear consensus in the literature defining the boundaries of the terms metallosis, ALVAL, and pseudotumors, and they used an umbrella term, adverse reaction to metal debris (ARMD), to describe arthroplasty failures associated with pain, a large sterile effusion of the hip, and/or macroscopic necrosis/metallosis.

Campbell et al. [6] advocated use of the ALVAL score, a 10-point histological score consisting of synovial lining integrity, inflammatory cell infiltrates, and tissue organization. The highest ALVAL scores occurred in patients who were revised for pain and suspected metal hypersensitivity. Other studies suggested that a cytotoxic response to metal wear debris could be the cause of ARMD [7–10]. In addition, patient susceptibility has been considered to be one of the major causes of pseudotumors [11].

Our hypothesis was that T cell-mediated type IV hypersensitivity could be a major cause of ARMD. This hypothesis was studied by lymphocyte stimulation testing and immunohistochemistry in patients with ARMD.

## Methods

### Patients

Thirteen consecutive patients with ARMD underwent revision surgery following metal-on-metal THA (15 hips) from December 2010 to March 2014 at our institution. The patient population consisted of one man and 12 women with a mean age of 68 (60 to 83) years and a mean body mass index of 24.9 (21.6 to 31.7) kg/m^2^. Cormet (Corin, Cirencester, UK) was used in 12 hips and Pinnacle (DePuy, Warsaw, IN) was used in three hips for the primary THA. Magnetic resonance imaging revealed pseudotumors in 11 patients (13 hips). Three hips were characterized as having the cystic type, one as the solid type, and nine as the mixed type. Two other patients underwent revision surgery due to symptomatic cup loosening. All hips had necrosis of periprosthetic soft tissues. The mean time to revision was 3 years and 7 months (1 year and 2 months to 6 years and 1 month). This study was approved by the ethics committee of Mie University, and all patients provided their informed consent.

### Lymphocyte stimulation testing

Lymphocyte stimulation testing was conducted before revision surgery according to the methods described by Niki et al. [12] at Mitsubishi Chemistry Medience Co, Ltd (Tokyo, Japan). Briefly, peripheral blood lymphocytes (PBLs) were separated from blood samples obtained from patients. PBLs were suspended with 10 % fetal bovine serum. A proliferative response to phytohemagglutinin was first confirmed to assume validity of the in vitro response and exclude drug or infective interference. Cultures consisting of 200 ml of PBL suspension were added to each well of a 96-well plate and incubated for 72 h in the presence of metal salts. The concentrations for maximal [3H]-incorporation into PBLs were determined. Neutralizing antibody against HLA-DR (clone, B8.12.2; Beckman Coulter, Marseille, France) was added at a final concentration of 5 mg/ml for 72 h of incubation. [3H] incorporation into lymphocytes was measured during the final 6 h of incubation, using a scintillation counter. The stimulation index was expressed as a percentage of medium control, and the Δ stimulation index was calculated. A stimulation index > 200 % was considered positive [12].

### Metal ion concentrations

In six unilateral THAs, serum cobalt and chromium ion concentrations were measured before and after revision surgery. Cobalt levels were assayed using inductively coupled plasma mass spectrometry (Perkin-Elmer SCIEX Elan 6100 DRC ICP-MS system; Perkin-Elmer Instruments, Norwalk, CT) at Mayo Medical Laboratories (Rochester, MN), and chromium levels were assayed using a graphite furnace atomic absorption spectrometer (Z-5700; Hitachi Ltd, Tokyo, Japan) with polarization-Zeeman absorption at Mitsubishi Chemistry Medience Co, Ltd (Tokyo, Japan). The detection limit for each ion was 0.2 μg/L [13].

### Histologic and immunohistochemical evaluations

The tissue specimens in patients who underwent revision were fixed in 10 % neutral buffered formalin prior to processing and embedding in paraffin wax. Sample sections 5-μm-thick were stained with hematoxylin and eosin and examined by light microscopy [13], and ALVAL scoring was performed. Sections were also analyzed with immunohistochemistry using antibodies to T cells (CD3; DAKO, Glostrup, Denmark) and B cells (CD20; DAKO) to characterize the immunophenotype. Sections 5-μm-thick were treated with superblock solution (Scytek Laboratories, Logan, UT) before incubation with antibodies to T cells and B cells overnight at 48 °C. After washing, sections were incubated in 0.3 % H_2_O_2_ in methanol for 15 min to block endogenous peroxidase activity. After being washed, sections were treated with peroxidase-conjugated antimouse IgG Fab0 (1:500 MBL, Nagoya, Japan) for 1 h, followed by color development with diaminobenzidine/H_2_O_2_ solutions. Light counterstaining with hematoxylin was performed to aid orientation.

The predominant lymphocyte was determined based on whether T cells or B cells were dominant. T-cell dominant hips were further analyzed using antibodies to T helper (Th) cells (CD4; DAKO) and T cytotoxic (Tc) cells (CD8; DAKO) to determine whether Th cells or Tc cells were dominant.

### Statistical methods

The Wilcoxon signed-rank test was used to test differences in serum cobalt and chromium ion concentrations before and after revision surgery. The Mann-Whitney U test was used to compare ALVAL scores between patients with and without positive lymphocyte stimulation tests. Fisher’s exact test and the Mann-Whitney U test were used to compare metallosis, serum metal ion levels before revision, pseudotumor size, and lymphocyte stimulation tests between the hips with dominant B cell and T cell infiltrations. Statistical significance was set at a *p* value less than 0.05.

## Results

Lymphocyte stimulation testing showed that the mean stimulation indices for nickel, cobalt, and chromium were 192 % (52 to 453 %), 108 % (47 to 306 %), and 94 % (44 to 149 %), respectively. Five patients were nickel-sensitive, and one patient was also cobalt-sensitive before revision. No reactivity to chromium was detected.

The mean cobalt levels were elevated to 4.4 (1.9 to 7.5) μg/L before revision and then decreased significantly to 0.8 (0.3 to 1.5) μg/L after revision (*p* = 0.028). The mean chromium levels dropped significantly from 3.0 μg/L (0.8 to 5.9 μg/L, before revision) to 0.7 μg/L (0.2 to 1.6 μg/L, after revision, *p* = 0.043). The mean ALVAL score was 7 points (5 to 9). The ALVAL score showed no significant difference between patients with and without positive lymphocyte stimulation tests (*p* = 0.575).

**Fig. 1:**
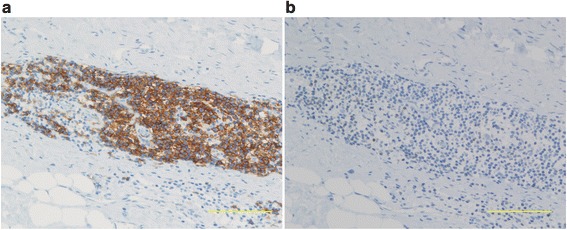
Immunohistochemical staining showing (**a**) numerous CD20-positive B cells and (**b**) scant CD3-positive T cell infiltration in periprosthetic tissue. Scale bar = 100 μm

Immunohistochemical studies showed that CD3-positive T cells were dominant in five hips with ARMD, and that CD20-positive B cells were dominant in 10 hips with ARMD (Fig. 1). Centrally placed B cell aggregates were loosely surrounded by T cells. In the five hips in which T cells predominated, CD8-positive Tc cells predominated in all five, and CD4-positive Th cells did not predominate in any hip. In four of five patients with positive lymphocyte stimulation tests, T cells were dominant, suggesting type IV hypersensitivity (Additional file 1: Table S1). Metallosis was found in nine hips (B-cell dominant five hips, T-cell dominant four hips, *p* = 0.580). The mean cobalt levels in the hips with dominant B cell and T cell infiltrations were 5.1 and 4.0 μg/L, respectively (*p* = 0.355). The mean chromium levels in the hips with dominant B cell and T cell infiltrations were 2.2 and 3.4 μg/L, respectively (*p* = 0.481). The sizes of pseudotumors ranged from 30 to 178 mm in diameter. The mean size of pseudotumors in the hips with dominant B cell and T cell infiltrations were 78 and 82 mm, respectively (*p* = 0.884). These results showed no significant differences between the hips with dominant B cell and T cell infiltrations. However, the hips with dominant T cell infiltration showed a higher incidence of a positive lymphocyte stimulation test (*p* = 0.017).

## Discussion

The diagnosis of metal hypersensitivity has not been fully established. Contact hypersensitivity to metal is a delayed type (Type IV) hypersensitivity reaction. Metal ions work as haptens. A hapten-carrier complex (antigen) is taken up by Langerhans cells (antigen-presenting cells: APCs) and then encountered by T cells [14]. T cells and, in particular, CD4-positive Th cells are responsible for shaping the immune response. Th cells are specific for antigens presented by major histocompatibility complex II (MHC II) molecules on the surface of APCs, while CD8-positive Tc cells are specific for the class I MHC molecules on non-body-own proteins. Tc cells can cause tissue damage and to a certain extent influence the local response [15, 16].

Testing for metal hypersensitivity has been historically conducted in vivo using skin patch testing. The patch test is considered the reference method for diagnosing contact allergy. The proportion of positive tests has increased significantly over time, especially in the last 20 years [17]. Thomas et al. [18] showed that, in 16 patients with revised metal-on-metal arthroplasty, patch test reactions were seen in 11/16 patients (69 %, 7/16 to cobalt, 7/16 to chromium, 4/16 to nickel); 10 of 16 patients (62 %) showed enhanced lymphocyte transformation test reactivity to metals (7/16 to nickel, 7/16 to cobalt, 5/16 to chromium). Nickel has long been identified as a cause of allergic dermatitis, affecting more than 10 % of patients. Cobalt sensitivity has been observed in approximately 1 % of the same population, with a significant degree of cross-reactivity between the two metals. In a European surveillance system for contact allergy in 2007–2008, the prevalence of nickel sensitivity was 12–27 %, with cobalt sensitivity in 5–14 % [19]. The patch test for metal hypersensitivity after arthroplasty is unreliable, because skin is an excellent barrier, sealing the immune system from direct environmental contact. A better surface for allergy testing would be a mucous membrane such as the oral cavity, but placement and maintenance of oral tests would be difficult, and would still not have the same environment of a joint. The inability to implant test material on the joint surface has led to the use of the in vitro lymphocyte stimulation test [17, 20, 21]. The lymphocyte stimulation test has been used to investigate metal sensitivity related to implant failure [9, 12, 22]. T cells play a crucial role in metal hypersensitivity reactions. In four patients with positive lymphocyte stimulation tests and dominant T cell infiltration, the cause of ARMD could be suggested to be type IV hypersensitivity. Nickel has been reported to be an offending metal for metal hypersensitivity [20, 21]. However, one might wonder if nickel exposure is relevant, since there is only a small percentage of nickel in cobalt chromium alloys [20]. Since B lymphocyte infiltration is not characteristic of a type IV hypersensitivity reaction [23], the major cause of ARMD could not be type IV hypersensitivity in the remaining nine patients. Kwon et al. [9] demonstrated that lymphocyte reactivity to cobalt, chromium, and nickel did not significantly differ in patients with pseudotumors compared to patients without pseudotumors. A T-lymphocyte-mediated type IV hypersensitivity reaction may not be the dominant biological reaction involved in the occurrence of pseudotumors.

Lymphoid structures are classified as primary lymphoid organs (thymus and bone marrow), secondary lymphoid organs that arise during embryonic development (lymph nodes, spleen, tonsil, Peyer’s patches), and tertiary lymphoid organs that arise in chronically inflamed adult tissue. Tertiary lymphoid organs are synthesized with microbial infections and autoimmune diseases, such as rheumatoid arthritis and Sjögren’s syndrome. Features associated with tertiary lymphoid organs are centrally placed B cell aggregates, loosely surrounded by T cells [24]. The B and T cell-containing aggregates seen in ARMD bear a remarkable resemblance to tertiary lymphoid organs that arise in chronically inflamed adult tissue. In addition to the well-described T cell-mediated type IV hypersensitivity response, an under-recognized immunological reaction to metal wear debris involving B cells and the formation of tertiary lymphoid organs could occur in a distinct subset of patients with ARMD [25]. The presence and importance of B cells have been largely underestimated in the cellular reaction to metal wear debris. The significance of B cells and tertiary lymphoid organ formation and their correlations with outcome require further evaluation. Further studies to elucidate the association between tertiary lymphoid organ formation and autoimmunity to metal hapten-carrier complexes and/or T cell-mediated inflammatory responses are needed to develop novel therapies to overcome ARMD [25].

This study has some limitations. First, a small number of patients were studied, and all patients but one were female. Second, the cause for revision was ARMD in all patients after metal-on-metal THA. No patients were revised because of dislocation and infection, and this study had no control group of tissues without ARMD. Immunohistochemical evaluations are warranted to compare the results between the patients with ARMD after metal-on-metal THA and without ARMD including the patients after non-metal-on-metal THA as a control group [26]. Third, the presence or absence of a metal hypersensitivity reaction has no implications regarding treatment. Further studies are needed to develop strategies for prevention and future treatment for ARMD.

## Conclusions

The cause of ARMD could be metal hypersensitivity in some patients; however, the majority of patients with ARMD had no evidence of metal hypersensitivity, and our hypothesis was not verified. Metal hypersensitivity was not the dominant biological reaction involved in the occurrence of ARMD.

### Ethics approval and consent to participate

This study was approved by the ethics committee of Mie University.

### Consent for publication

All patients provided their informed consent.

### Availability of data and materials

We had full access to all of the data in the study and take responsibility for the integrity of the data and the accuracy of the data analysis. The data is available on request from the corresponding author.

## Additional file

Additional file 1: Table S1. Lymphocyte stimulation test and dominant lymphocytes. (XLSX 9 kb)

## Abbreviations

ALVAL: aseptic lymphocyte-dominated vasculitis-associated lesion
ARMD: adverse reaction to metal debris
THA: total hip arthroplasty

## References

[1] Kusaba A, Kondo S, Mori Y, Kuroki Y (2008). In vivo change of elastic property in polyethylene acetabular components. Mod Rheumatol.

[2] Pandit H, Glyn-Jones S, McLardy-Smith P, Gundle R, Whitwell D, Gibbons CL (2008). Pseudotumours associated with metal-on-metal hip resurfacings. J Bone Joint Surg [Br].

[3] Daniel J, Holland J, Quigley L, Sprague S, Bhandari M (2012). Pseudotumors associated with total hip arthroplasty. J Bone Joint Surg Am.

[4] Willert HG, Buchhorn GH, Fayyazi A, Flury R, Windler M, Köster G (2005). Metal-on-metal bearings and hypersensitivity in patients with artificial hip joints. A clinical and histomorphological study. J Bone Joint Surg Am.

[5] Langton DJ, Jameson SS, Joyce TJ, Hallab NJ, Natu S, Nargol AV (2010). Early failure of metal-on-metal bearings in hip resurfacing and large-diameter total hip replacement: a consequence of excess wear. J Bone Joint Surg [Br].

[6] Campbell P, Ebramzadeh E, Nelson S, Takamura K, De Smet K, Amstutz HC (2010). Histological features of pseudotumor-like tissues from metal-on-metal hips. Clin Orthop Relat Res.

[7] Mahendra G, Pandit H, Kliskey K, Murray D, Gill HS, Athanasou N (2009). Necrotic and inflammatory changes in metal-on-metal resurfacing hip arthroplasties. Acta Orthop.

[8] Kwon YM, Glyn-Jones S, Simpson DJ, Kamali A, McLardy-Smith P, Gill HS (2010). Analysis of wear of retrieved metal-on-metal hip resurfacing implants revised due to pseudotumours. J Bone Joint Surg [Br].

[9] Kwon YM, Thomas P, Summer B, Pandit H, Taylor A, Beard D (2010). Lymphocyte proliferation responses in patients with pseudotumors following metal-on-metal hip resurfacing arthroplasty. J Orthop Res.

[10] Hasegawa M, Yoshida K, Wakabayashi H, Sudo A (2012). Pseudotumor with dominant B-lymphocyte infiltration after metal-on-metal total hip arthroplasty with a modular cup. J Arthroplasty.

[11] Matthies AK, Skinner JA, Osmani H, Henckel J, Hart AJ (2012). Pseudotumors are common in well-positioned low-wearing metal-on-metal hips. Clin Orthop Relat Res.

[12] Niki Y, Matsumoto H, Otani T, Yatabe T, Kondo M, Yoshimine F (2005). Screening for symptomatic metal sensitivity: a prospective study of 92 patients undergoing total knee arthroplasty. Biomaterials.

[13] Hasegawa M, Yoshida K, Wakabayashi H, Sudo A (2012). Cobalt and chromium ion release after large-diameter metal-on-metal total hip arthroplasty. J Arthroplasty.

[14] Kaplan DH (2010). In vivo function of Langerhans cells and dermal dendritic cells. Trends Immunol.

[15] Gao GF, Jakobsen BK (2000). Molecular interactions of coreceptor CD8 and MHC class I: the molecular basis for functional coordination with the T-cell receptor. Immunol Today.

[16] Landgraeber S, von Knoch M, Löer F, Brankamp J, Tsokos M, Grabellus F (2009). Association between apoptotis and CD4(+)/CD8(+) T-lymphocyte ratio in aseptic loosening after total hip replacement. Int J Biol Sci.

[17] Granchi D, Cenni E, Giunti A, Baldini N (2012). Metal hypersensitivity testing in patients undergoing joint replacement: a systematic review. J Bone Joint Surg [Br].

[18] Thomas P, Braathen LR, Dörig M, Auböck J, Nestle F, Werfel T (2009). Increased metal allergy in patients with failed metal-on-metal hip arthroplasty and peri-implant T-lymphocytic inflammation. Allergy.

[19] Uter W, Aberer W, Armario-Hita JC, Fernandez-Vozmediano JM, Ayala F, Balato A (2012). Current patch test results with the European baseline series and extensions to it from the 'European Surveillance System on Contact Allergy' network, 2007-2008. Contact Dermatitis.

[20] Thomas P, Stander S, Stauner K, Schraml A, Banke IJ, Gollwitzer H (2013). Arthroplasty patients and nickel sensitization: What do patch test and lymphocyte transformation test tell us. Semin Arthroplasty.

[21] Hallab N, Merritt K, Jacobs JJ (2001). Metal sensitivity in patients with orthopaedic implants. J Bone Joint Surg Am.

[22] Hallab NJ, Anderson S, Stafford T, Glant T, Jacobs JJ (2005). Lymphocyte responses in patients with total hip arthroplasty. J Orthop Res.

[23] Nasser S (2007). Orthopedic metal immune hypersensitivity. Orthopedics.

[24] Aloisi F, Pujol-Borrell R (2006). Lymphoid neogenesis in chronic inflammatory diseases. Nat Rev Immunol.

[25] Mittal S, Revell M, Barone F, Hardie DL, Matharu GS, Davenport AJ (2013). Lymphoid aggregates that resemble tertiary lymphoid organs define a specific pathological subset in metal-on-metal hip replacements. PLoS One.

[26] Fujishiro T, Moojen DJ, Kobayashi N, Dhert WJ, Bauer TW (2011). Perivascular and diffuse lymphocytic inflammation are not specific for failed metal-on-metal hip implants. Clin Orthop Relat Res.

